# Between guidelines and reality: expert neurologists’ perspectives on structural resources for ALS care in Germany

**DOI:** 10.1186/s42466-026-00481-9

**Published:** 2026-04-08

**Authors:** Katharina Linse, Dorothée Lulé, Florian Schöberl, Peter Reilich, Benjamin Ilse, Moritz Metelmann, Clemens Eickhoff, Sarah Bublitz, Stefan Lorenzl, Matthias Boentert, Susanne Petri, Annekathrin Rödiger, Uta Smesny, Joachim Wolf, Ute Weyen, Daniel Zeller, Johannes Dorst, André Maier, Thomas Meyer, Paul Lingor, Andreas Hermann, Martin Regensburger, Julian Großkreutz, Clemens Runge, Hanna Sophie Lapp, Maren Freigang, Maximilian Vidovic, Elisa Aust, Constanze Weber, René Günther

**Affiliations:** 1 Department of Neurology, Faculty of Medicine and University Hospital Carl Gustav Carus, TUD Dresden University of Technology, Fetscherstraße 74, 01307 Dresden, Germany; 2 Department of Neurology, Faculty of Medicine, Ulm University, Oberer Eselsberg 45, 89081 Ulm, Germany; 3 Department of Neurology with Friedrich-Baur-Institute, University Hospital, Ludwig-Maximilians-University (LMU), Marchioninistrasse 15, 81377 Munich, Germany; 4 Department of Neurology, Jena University Hospital, Am Klinikum 1, 07747 Jena, Germany; 5 Department of Neurology, University Medical Center Leipzig, Liebigstrasse 20, 04103 Leipzig, Germany; 6 Department of Neurology and Clinical Neurophysiology, Helios MVZ Kassel, Bergmannstrasse 30-32, 34121 Kassel, Germany; 7 Department of Neurology and Palliative Care, Agatharied Hospital, Norbert- Kerkel-Platz, 83734 Hausham, Germany; 8 Institute of Palliative Care, Institute of Nursing Science, Paracelsus Medical University, Salzburg, Austria; 9 Neuromedical Centre, Department of Sleep Medicine, Klinikum Osnabrück, Am Finkenhügel 1, 49076 Osnabrück, Germany; 10 Department of Neurology Institute of Translational Neurology, Faculty of Medicine, University of Münster, Albert-Schweizer-Campus 1, 48149 Münster, Germany; 11 Diakovere Hospital, Anna-von-Borries-Strasse 1-7, 30625 , Hannover, Lower Saxony Germany; 12 Department of Neurology, Hannover Medical School, Carl-Neuberg-Strasse 1, 30625 Hannover, Germany; 13 Center for Rare Diseases, Jena University Hospital, Am Klinikum 1, 07747 Jena , Germany; 14 Brüderklinikum Julia Lanz, Speyerer Strasse 91-93, 68163 Mannheim, Germany; 15 Department of Neurology, BG University Hospital Bochum, Bürkle de la Camp- Platz 1, 44789 Bochum, Germany; 16 Department of Neurology, Würzburg University Hospital, Josef-Schneider- Strasse 11, 97080 Würzburg, Germany; 17 Department of Neurology, Charité – Universitätsmedizin Berlin, Augustenburger Platz 1, 13353 Berlin, Germany; 18 Klinikum rechts der Isar, TUM School of Medicine and Health, Technical University of Munich, Ismaninger Strasse 22, 81675 Munich, Germany; 19 German Center for Neurodegenerative Diseases Munich, Feodor-Lynen-Strasse 17, 81377 Munich, Germany; 20 Translational Neurodegeneration Section Albrecht Kossel, University Medical Center Rostock, Gehlsheimer Strasse 20, 18147 Rostock, Germany; 21 German Center for Neurodegenerative Diseases Rostock/Greifswald, Gehlsheimer Strasse 20, 18147 Rostock, Germany; 22 Department of Molecular Neurology, Erlangen University Hospital, Schwabachanlage 6, 91054 Erlangen, Germany; 23 Precision Neurology of Neuromuscular and Motor Neuron Diseases, University of Lübeck, Ratzeburger Allee 160, 23538 Lübeck, Germany; 24 German Center for Neurodegenerative Diseases Dresden, Tatzberg 41, 01307 Dresden, Germany; 25 Saxon Hospital Arnsdorf, Hufelandstrasse 15, 01477 Arnsdorf, Germany

**Keywords:** Amyotrophic Lateral Sclerosis, Multidimensional care, Palliative care, Motor neuron disease, Psychosocial care, Healthcare system

## Abstract

**Background:**

Amyotrophic lateral sclerosis (ALS) is a fatal motor neuron disease, leading to an inexorable decline in voluntary muscle function, and finally to death within 2–4 years. The provision of professional ALS care is a multifaceted and continually evolving challenge, including the management of symptoms, advanced care planning, and the provision of psychosocial support. The core objective is to minimize suffering by optimizing symptom management and preserving quality of life. European and German guidelines recommend a specialized, multidisciplinary, and multiprofessional team, including collaborations with palliative care providers. While this is a validated approach to ensure optimal care and patient satisfaction, real-world experience suggests that the German healthcare system may not fully meet these requirements.

**Methods:**

This study assessed resources of specialized ALS centres in Germany, focusing on the structural prerequisites for the provision of multidimensional care and collaboration with specialised palliative care (SPC) providers. A mixed methods design was used, comprising remote video interviews with neurologists specialized in ALS, including standardised questions and an open section.

**Results:**

Sixteen neurologists representing their ALS centres were interviewed. The findings indicated a substantial discrepancy in the allocation of time and personnel resources among the centres. The majority of interviewees regarded resources to be inadequate and reported deficiencies in multidisciplinarity and networking. Consequently, certain components of ALS care - particularly psychosocial concerns - have been documented as being occasionally disregarded due to limitations in time or human resources. A number of interviewees expressed criticism regarding the inadequate access to and suboptimal collaboration with SPC providers. The compensation for patient care and interprofessional communication was not perceived as cost-effective.

**Conclusions:**

Our results suggest that limited resources may prevent the provision of guideline-based care for people living with ALS and their families, even in specialized outpatient clinics. To facilitate the delivery of comprehensive care by ALS centers throughout the entire disease course, the establishment of operational standards concerning their multi-professional staffing and adequate compensation is imperative. Further research is needed to develop feasible concepts of how specialized neurological palliative care can be made reliably accessible to all patients in need.

**Supplementary Information:**

The online version contains supplementary material available at 10.1186/s42466-026-00481-9.

## Introduction

Amyotrophic lateral sclerosis (ALS) is an adult-onset motor neuron disease, currently affecting over 5,000 people in Germany [1]. Due to the progressive degeneration of motor neurons, ALS leads to a continuous decline in voluntary muscle function, resulting in significant immobility as well as disabilities of speech and swallowing. Respiratory muscle weakness is often inevitable and results in chronic ventilatory failure that usually leads to premature death within 2–4 years from symptom onset, unless people living with ALS (plwALS) opt for long-term invasive ventilation [2].

The requirements for professional ALS care are complex and vary dynamically during the course of the disease. The overriding goal is to minimise symptom load and maintain the best possible quality of life (QoL) [3]. In addition to disease-modifying therapy with riluzole and tofersen (for patients with a confirmed (likely) pathogenic mutation in the *superoxide dismutase 1 (SOD1)* gene), and optimized medical symptom management, current treatment guidelines emphasize numerous further tasks [3, 4]. These include advance care planning (ACP), the consideration of psychosocial and existential needs, initiation of adequate supply with medical aids and remedies, and the integration of palliative care:

ACP, predicated on a shared decision-making process, is regarded as the gold standard in both palliative medicine and ALS care [5]. The overarching objective is to ensure patient autonomy, which is one of the four fundamental principles of medical ethics [6]. Among other aspects, the potential and limitations of treatment for respiratory insufficiency must be discussed. Most plwALS request assistance and counsel for drawing up an advance healthcare directive or appointing a healthcare proxy [7]. These conversations require careful timing, along with sensitivity to each patient’s level of knowledge, strain thresholds, and available resources [3, 4, 8].

Adequate provision with medical aids and remedies constitutes a further essential component of ALS care. These measures have the potential to compensate for at least some of the physical deficits, thereby enhancing patients’ autonomy, participation, and QoL [9, 10].

In addition, the fatal prognosis of ALS, in conjunction with the progressive loss of autonomy, often lead to a high level of psychosocial and existential suffering for both plwALS and their families. A substantial proportion of plwALS and their family caregivers exhibit symptoms of depression and anxiety that necessitate treatment, as indicated by findings from both national and international studies [11, 12]. Patients’ QoL is more strongly correlated with their psychological, rather their physical condition [13, 14]. In some plwALS, psychosocial distress may even lead to a desire for hastening death [15]. In caregivers, distress may result in a high level of caregiver burden and negative health consequences, which can even persist for years after the patient’s death [16, 17]. The need for psychosocial support for both groups is obvious and well-known, although the number of studies on specific interventions is sparse [18–22].

As the symptom load increases - particularly in the final stages of the disease - additional medical, sociomedical, and psychosocial tasks arise. In case of high and complex symptom load, additional support by providers of specialised palliative care (SPC) is recommended [4]. A heuristic concept for the timing of its initiation is the 12-month-surprise question – “Would I be surprised if this patient were to die within the next 12 months?” – however, SPC may also be necessary from the point of diagnosis [23, 24]. In Germany, integrating specialised outpatient palliative care is the most common option in practice; referral to hospices or palliative care units are additional options, especially in advanced disease stages [25]. According to the definition of the WHO, providing ‘palliative care’ involves not only the care of the patient, but also of their families [26].

In line with these complex, multidimensional requirements, European and German medical guidelines clearly recommend the provision of comprehensive ALS care by a specialised, multidisciplinary and multiprofessional team, including providers from the field of SPC [3, 4, 27–29]. Both aspects – multidimensionality of care and the inclusion of SPC – are associated with improved symptom management, higher patient satisfaction, and better QoL (for detailed evidence, see the guidelines). Fundamentally, professional, needs-based ALS care requires sufficient time.

However, real-world experience suggests discrepancies between the current framework conditions for standard medical care in Germany and the necessities of multidimensional neurological, psychosocial, and palliative care for plwALS and their caregivers. Non-medical services, including but not limited to psychological support and social counselling, are not consistently incorporated into ALS care or reimbursable under the current system. Furthermore, there is an absence of established standards for the integration of SPC. The ability to meet the multidimensional needs of those affected throughout the entire course of the disease under these circumstances is questionable. However, systematic evaluation of deficiencies in ALS care is lacking. The present analysis of the perspectives of professional care providers intends to identify structural shortcomings and to contribute to the improvement of multidisciplinary and palliative ALS care in Germany.

## Methods

Staffing and time resources in specialised ALS centres in Germany were systematically assessed. Furthermore, collaborative structures and networking with SPC providers as well as subjectively perceived structural deficits were investigated.

For this purpose, a mixed methods design was used; quantitative and qualitative data were collected in parallel, in order to gain a more comprehensive understanding by complementing and validating both data sources. We conducted interviews with neurologists working in ALS centres via video telephony. These interviews consisted of two parts: a standardised section with predefined questions and answer options, to descriptively assess quantifiable aspects of local ALS care structures, subjective shortcomings and potential solutions (see supplementary material). The second section contained an introductory question with an open response format; the participants were asked about their demands for structural improvements in ALS care, in addition to or as a summary of those asked about in the first part. Manual conventional content analysis was used to study and interpret these qualitative data [30]: The interviewees’ responses were transcribed and broken down into meaning units. These were analysed in terms of their content and assigned preliminary labels. In a further analytic cycle, these assignments were reviewed to ensure that they adequately captured the participants’ representations, and final categories were defined.

Between January and March 2025, 17 neurologists of German ALS centres who had previously collaborated in a large patient survey on psychosocial and palliative care in ALS [31] were invited to participate in the study. Answers from sixteen interviewees were recorded. The interviews were carried out by an experienced researcher (KL) and lasted between 20 and 50 min. At the beginning of the interview, the aims and procedural flow were explained. After consent had been obtained, the interviews were recorded in order to enable a precise content analysis of the qualitative part. Questions and clarifications were possible at any time. Basic characteristics of the participants and the assigned centres are displayed in Table 1.

**Table 1:** Characteristics of the Interviewees and the assigned ALS centres

Centre-ID	Characteristics of the ALS centres			Characteristics of the Interviewees		
	plwALS cared for per year	Physician positions officially proposed for ALS care		ALS care since years	ALS patient contacts per week*	Additional qualification in Palliative Medicine
**1**	50–200	0.50		10.0	25	No
**2**	50–200	0.20		20.0	8	No
**3**	50–200	0.00		17.0	4	Yes
**4**	50–200	0.90		20.0	11	Yes
**5**	50–200	0.25		6.0	10	Yes
**6**	50–200	1.60		16.0	13	Yes
**7**	> 200	0.75		9.0	17	No
**8**	50–200	0.00		19.0	7	No
**9**	50–200	0.25		15.0	6	No
**10**	< 50	0.25		9.0	4	No
**11**	> 200	1.00		0.8	23	No
**12**	> 200	1.00		20.0	5	No
**13**	> 200	3.00		21.0	17	No
**14**	> 200	2.30		19.0	7	Yes
**15**	50–200	0.70		11.0	10	Yes
**16**	> 200	0.45		9.0	10	Yes

* in addition to in-person visits, this may also include video or telephone consultations

**Table 2:** Resources available for ALS care in the centres

Centre-ID	Professional groups involved in ALS care in the respective centre (other than neurologist)				Neurologist		Resources in sum considered sufficient?*				Provision of end-of-life care	
	Organisational tasks	Provision of care	Time/patient (min)	Time/visit^+^ (min)	Time/patient (min)	Time/visit^+^ (min)	pPerson-nel	Time	Reim-bursement		Cooperation with SPC **	Care until eol
**1**	Nurse(s)	Nurs(es)	5	20	60	75	-^1^	-^1^	-		2	No
**2**	Nurse(s) Social worker^D^	Nurs(es) Social worker^D^	30	60	30	60	-^1^	+^1^	-		2	Yes
**3**	Nurse(s) Case manager Secretary	Case manager Neuropsychologist Social worker (voluntary)	45	65	60	90	+^1^	+^1^	-		2	Yes
**4**	Nurse(s) Social worker^D^	Social worker^D^ Medical supply consultant ^D^	90	105	75	105	+	+	+		1	No
**5**	Nurse(s)	Social worker^D^ Medical supply consultants^D^	60	90	45	75	+^1^	+^1^	-		1	Yes
**6**	Nurse(s)	Nurs(es)	15	30	45	75	-	-	-		3	No
**7**	Nurse(s) Case manager Social worker	Case manager Social worker Palliative care outpatient clinic	30	90	45	60	+^1^	+	-		2	No
**8**	Nurse(s)	Nurs(es) Palliative care consultancy service	30	60	60	97	-^1^	+	-		2	No
**9**	Nurse(s) Social worker^D^	Social worker^D^ Medical supply consultant ^D^ Palliative care consultancy service	45	60	25	30	+^1^	+^1^	-		1	No
**10**	Nurse(s) Study nurse	Social worker^D^ Medical supply consultant ^D^	30	75	60	105	+^1^	+	-		2	Yes
**11**	Nurse(s) Case manager^D^ Social worker^D^	Nurs(es) Case manager^D^ Social worker^D^ Therapists	45	105	60	105	-	+	Not known		1	No
**12**	Nurse(s) Social worker^D^	Nurs(es) Social worker^D^ Medical supply consultant ^D^ Palliative care consultancy service	30	60	90	120	+	-	-		3	No
**13**	Nurse(s) ALS specific care and case management network and platform	Nurs(es) ALS specific care and case management network and platform	60	120	60	90	-	-	-		2	Yes
**14**	Nurse(s) Study Nurse Medical documentarist	Therapists Medical supply consultant	30	50	45	74	-^1^	+^1^	-		0	Yes
**15**	Nurse(s)	Nurs(es) Social worker Pastoral worker	60	75	60	90	-^1^	+^1^	-		2	No
**16**	Nurse(s)	-	0	15	60	90	-^1^	-^1^	-		1	No
	**Median resp. Mode**		**30**	**60**	**60**	**90**	**-** ^**1**^	**+** ^**1**^	-		**2**	**No**

^D^employed by the German Society of Muscle Diseases (Deutsche Gesellschaft für Muskelkranke e.V.), a national self-help organization; eol, end-of-life; SPC, providers of Specialised Palliative Care

^+^ total time per patient visit = time spent with the patient plus preparation and follow-up time

* “Are resources in your centre sufficient, in view of the complex needs of patients?”

- = no

+ = yes

^1^ = we rely on resources which are originally not intended for ALS care** “Are there any established partnerships with SPC providers at your centre?”0= no, not at all1= no, only very loose contacts, I simply recommend patients to contact these services.2= yes, there is an informal cooperation, I refer patients to these services and we exchange medical reports3= yes, a close cooperation is established, including case conferences/telephone calls, if necessary

## Results

### Quantitative results

#### Resources

All centres reported that nursing staff provided support with organizational tasks related to ALS patient consultations, including appointment scheduling and the collection of previous medical reports. Further professional groups were involved in 62% of the centres (see Table 2). Regarding support from other professional groups for the specialised co-care of plwALS, there was a large variance regarding their availability (ranging from no staff available at all to four other professional groups) and their time resources per patient and visit. The time per patient and visit available to the neurologist also varied greatly between the centres, with a range of 65 min. On average, 60 min were available for the consultation and a further 30 min for preparation and post-appointment follow-up. In sum, most neurologists considered the personnel resources to be inadequate in view of the complex, dynamic, multidimensional needs of their patients - even when personnel originally not intended for ALS care was employed. The personnel resources were only considered sufficient by centres that were able to access other professional groups beyond nurses for the co-care of patients on demand. This included recourse on personnel not planned for ALS care, or personnel not employed by the clinic, but by a case management platform or a patient organisation. Two thirds (69%, *N* = 11) of the centres perceived their time resources as sufficient, however, this involved recourse to resources originally not intended for ALS care in slightly more than half of these centres (6 out of 11). In relation to their perceived care responsibilities, five interviewees considered the available time per patient insufficient. This primarily involved centres with objectively limited time per patient, which was also associated with limited access to other professional groups beyond nursing staff (Table 2). In 14 out of 16 centres, compensation was perceived as not cost-effective, regarding the time and effort required. One interviewee could not make a reliable statement on this.

## Subjective shortcomings and suggested improvements

**Fig. 1:**
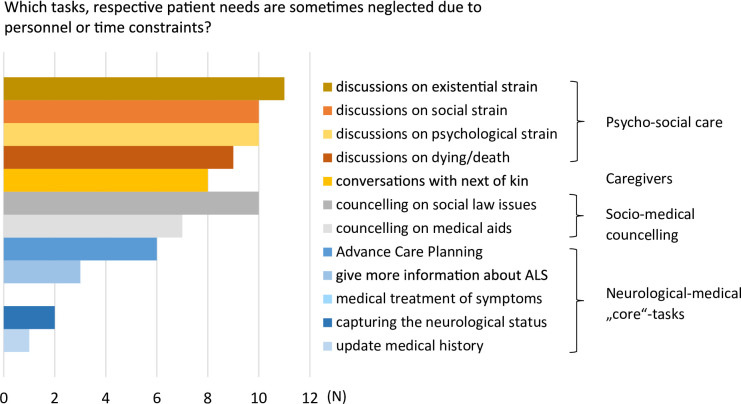
Frequency of interviewees’ answers to the question, which tasks or patient needs are sometimes neglected due to personnel or time constraints

When asked if some tasks, in relation to the specific needs of each patient, were sometimes neglected due to personnel or time constraints, all but one interview partner admitted to experiencing this at least occasionally. Considering the neurological-medical core tasks, this never applied to the medical treatment of symptoms but did apply to ACP in six cases (Fig. 1). Two thirds of the interviewees felt that resources were insufficient for socio-medical tasks such as providing adequate advice on assistive medical aids or social law issues. Psychosocial issues were reported to be inadequately addressed, with only two centres reported no shortcomings in this area at all. Considering the integration of SPC in ALS care, more than half of the participants stated that some kind of cooperation with the corresponding providers was established in their centre. However, cooperations were mostly described as informal and loose without standardised paths or rules (Table 2).

**Fig. 2:**
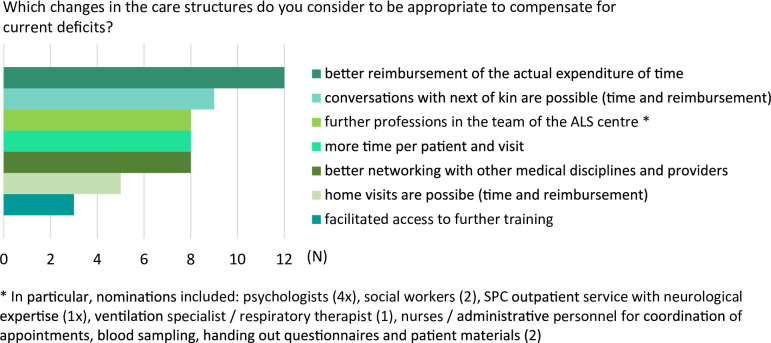
Frequency of interviewees’ answers to the question, which changes in the care structures are considered to be appropriate to compensate for current deficits

When asked about appropriate changes in the care structures to compensate for current deficits ALS care, the most frequent responses referred to an improvement of financial resources, and consequently personnel and time resources (Fig. 2). Additionally, half of the interviewees considered a more multiprofessional team as well as more liable and intensive networking with external medical specialties and providers relevant.

## Qualitative results

Analysis of the answers identified four overarching categories (Table 3):

**Table 3:** Interviewees answers in the open section of the interview, referring to the question on the most needed structural changes in ALS care

Category	Subcategory	Example statement	Centre-ID
Resources	Time	- *Questions about the end of life*,* about dying*,* these are themes we often do not talk about in the end*,* because we simply do not have a reasonable time framework to deal with that properly.*	10
	Personnel	- *We could make appointments much earlier if there was an assistant physician who would do the repeat visits*,* and someone who would take over non-medical tasks*,* e.g. blood sampling*,* handing out questionnaires*	16
		- *So in my case*,* it is a one-person-show (…)*,* ultimately there is no position planned for the outpatient clinic at all*	8
	Remuneration	- *I cannot have conversations with the next of kin*,* because they are not reimbursed*	16
		- *(referring to the need to standardize which professions belong to the multi-professional team of an ALS centre:) (…) which of course would have to be reflected in the reimbursement*,* otherwise nobody can afford and implement that*	13
		- *(following the statement*,* that time resources per patient and visit is perceived sufficient:) … of course*,* this is not funded in any way.*	8
Multidisciplinarity/Networking	General issues	- *What we lack here is multi-professionalism: better networking with nutritional counseling*,* outpatient palliative care services*,* palliative care units.*	10
		- *What is needed are psychologists*,* social workers*,* medical aid counsellors – currently (…) the neurologists themselves just try to cover these issues as good as they can*	6
		- *I think a multidimensional concept is very important*,* in order to be able to cover all the issues that the patient brings with them*,* the issues that concern and burden them. Regardless of who (which professional group) does what exactly*,* above all*,* you need a lot of time so that the patient feels acknowledged as a person*,* and that we can support them well with this illness.*	11
	Other medical disciplines	- *Getting appointments for pulmonary diagnostics or sleep laboratory is often difficult*,* despite personal contacts; appointments are often delayed due to staff shortages*	2
		- *there is a lack of medical multidisciplinarity*,* so one would like to have closer contact with other disciplines*,* especially pulmonologists (…)*,	5
	Self-help	- …*it’s important that we agree on a course of action*,* so that (…) no treatments are recommended that we*,* as medical professionals*,* would actually not recommend. We should talk with a unanimous voice.*	12
		- *Cooperation with self-help groups must be more regular. It has happened that medical decisions*,* such as the initiation of ventilation*,* were made without my knowledge*,* the patient was sent to another clinic*,* and I only got notice about it at the next follow-up appointment.*	4
	Specialised Palliative Care	- *Better cooperation in terms of information exchange with SAPV teams would be desirable. A good competence in relation to ALS is not available in all teams*,* as they (…) mostly care for oncological patients*,* so I would wish for more cooperation and exchange.*	4
		- *(…)*,* better networking with the palliative care team*,* palliative care unit*,* palliative care outpatient clinic in our house. Unfortunately*,* there is no close contact because they are very focused on oncological issues.*	5
		- *More networking with (…) volunteers would be good*,* outpatient hospice services can also bring a relief for patients and families*,* as companions*,* especially when things are going wrong within the family*	7
	Psychological/Pastoral Support	- *…also psychotherapists: I had a woman here just yesterday who received the diagnosis and was so upset*,* (…) all you can do is refer her to the hotline of the Association of Statutory Health Insurance Physicians*,* (…)*	10
		- *and what is a major deficiency is psychological support for patients and their families for this special situation*	5
		- *What we do (…) that’s a bit of symptom control*,* but that’s not the biggest thing*,* but rather the accompanying*,* the psychosocial accompanying of these existential issues*,* including assisted suicide*,* which is often addressed by the patients*,* accompanying the caregivers*,* advance planning (…) - Psychosocial issues that don’t always need to be accompanied by a doctor.*	15
		- *The pastoral counsellor who then joined the team was a great asset to the support*,* he has a completely different role (…) the spiritual needs of the patients are very similar*,* regardless of faith (…)*,* feeling connected to nature*,* having a closer relationship with the family*,* (…) the spiritual needs of the caregivers are equal*,* perhaps even much greater (…) that is why I think the inclusion of pastoral care can bring in a completely different level*,* (…) psychologists have very similar areas of responsibility*,* talk about similar issues.*	15
		- *(…)*,* psychologists would be good*,* we often discuss how we could organize that*,* how we could finance it*	11
Standardization of care paths and structures	Referring to the outpatient clinics	- *According to the guideline*,* the multi-professional team comprises 11 professions from diagnosis to death (…)*,* but in not a single outpatient clinic*,* all of them are available*,* each has its own focus (…)*,* so a certain degree of standardization would be desirable.*	13
		- *All university-associated outpatient clinics nationwide should be permitted to conduct video consultations with patients they already know*,* for example*,* to fill prescriptions on this basis. Means that the ventilated quadriplegic doesn’t have to come to me in person. However*,* there’s no nationwide solution; at this point*,* everyone is just ploughing his own furrow.*	6
		- *I think that specialized outpatient clinics should fundamentally have a special status*,* so that if we prescribe medical aids*,* it should be accepted by the payers. The same applies to new therapies or off-label therapies (…); my wish for the future would be that in such specialized centres*,* I would like it to be accepted that what is prescribed is really necessary. Same with regard to the degree of disability*,* for example. For me*,* this has to come with some kind of quality criteria for the centers*,* e.g. a social worker is mandatory part of the teams and so on. This would provide real psychological relief for patients and us alike*,* preventing frustration from building up. Criteria would have to be negotiated with politicians (…)*	3
		- *Or establish a kind of extensive inpatient complex treatment*,* where breathing*,* nutrition*,* patient directives*,* everything is taken into account*,* (…) family counselling*,* applications for medical aids*,* perhaps even applying for rehabilitation*,* and psychosocial-palliative care is initiated or intensified.*	3
	Patient journeys	- *It makes a big difference for them to be cared for by a team that is familiar with ALS*,* not just from a medical point of view. It was such a feeling of security for the people*,* not only for the patients*,* but also for their families*,* that they know there is someone they can always call and ask questions*,* and they know what they are talking about*,* they have cared for many ALS patients.*	15
		- *(On the subject of networking with other disciplines:) It’s more of a matter of assignment of patients; there’s no fixed network*,* no fixed cooperation*,* or a case manager who coordinates it all in some way.*	10
	Availability of Specialised Palliative Care	- *The core issue is that people can no longer come at some point*,* but still have a high need*,* and then some are not yet accepted by SAPV – this varies from team to team – to fill this gap*	15
		- *SAPV was rejected by the health insurance company because she has been tracheotomized*,* so she has decided “for life” and then supposedly does not need SAPV*,* that is so ridiculous - probably it is only about the costs*	3
		- *The problem is that one can no longer get to the outpatient clinic due to lost mobility (…) this can create a gap because there is simply no longer any specialist (neurological) medical support available. The SAPV team is very knowledgeable in many areas*,* but not everyone regularly cares for ALS patients and is therefore so familiar with symptomatic therapies.*	9
		- *ALS homecare* [specialized palliative homecare service for plwALS] *should be available nationwide*,* there is already a gap*,* sometimes there is an undersupply in the critical phase*,* when the patient can no longer physically come to us*,* but the SPAV does not accept him yet because they say it is still “too early”*,* and do not see themselves as being responsible.*	4
		- *…or the SAPV-team rejects patients if they have not yet decided on ventilation*	7
		- *It would be desirable to standardize the cost coverage for SAPV*,* as there is already regular disagreement that they (health insurances) state*,* there was no acute need*	7
Overriding health policy issues	Supply with medical aids	- *What is often dramatic for patients and caregivers is the length of time it takes for adequate supply with medical aids*,* because the health insurance companies*,* despite repeated opposition*,* do not provide urgently needed medical aids*,* do not cover the costs*	2
		- *The provision with medical aids is also often difficult. (Do you mean delays or rejections?) Yes*,* both.*	10
	remedies	- *Outpatient supply with remedies therapies is sometimes problematic*,* both in terms of general capacities and those who are familiar with ALS*	10
		- *What is also a bit of a shortage are actually speech therapists*,* physiotherapists and occupational therapists that like to work with ALS patients and also make home visits.*	5
	Psychologists	- *(Regarding the availability of psychologists/psychotherapists:) …but that is generally difficult*,* even with Huntington etc.*,* if there are no acute suicidal tendencies*,* simply because of the capacities we have no offer to find a contact person for the patients in a timely manner*	10
	Remuneration of established practitioners	- *Care by established neurologists is often completely inadequate*,* which I consider to be a major problem*,* (…) because they (ALS patients) take up a lot of time*,* (…) there has to be the possibility of taking the high time requirement due to the severity of the illness into account in the corresponding billing catalogues*	6
	Compensation of “speaking medicine”	- *Speaking medicine is generally not sufficiently remunerated. One could save a lot of money elsewhere for sometimes aimless*,* redundant medical high-tech procedures*,* and instead invest it in speaking medicine*,* i.e. in dealing with human*,* individual issues (…) which are crucial for well-being*,* especially in the case of such an illness*	13

SAPV, specialized outpatient palliative care (Spezialisierte Ambulante Palliativversorgung)

### Resources

Representatives from five centres in this section complained about a shortage of time, financial, and personnel resources. Some interviewees reiterated their statement from section one, that ALS care is at least partially contingent on resources that are not actually intended for this purpose, and that despite that, not all needs of plwALS and their families can be adequately met, due to a paucity of overall resources. One interviewee exemplarily stated: *“ALS care needs improvement in all areas. I don´t think*,* that any centre in Germany can provide optimal support to the patients in exactly the way they need it. This isn´t covered financially*,* nor in terms of staff; there is a lack of structures (…)*,* and no psychological support. In my view*,* there are shortages everywhere*,* we´re trying to make it possible somehow with limited manpower. The entire system in Germany stands and falls with the commitment of those involved in the individual centres. This is absolutely not comparable to oncological care; it’s lagging massively behind. I don’t know how we can achieve a similarly well-defined care structure as for cancer patients. Perhaps the patient population is too small*,* or ALS doesn’t have a strong lobby. I’m not satisfied with the way things are.”* (Centre-ID 8).

## Multidisciplinarity/networking

Responses assigned to this category encompassed both the acknowledgement of the significance of multidisciplinarity in ALS care and the imperative for its reinforcement. Specifically, the necessity for close networking with other medical disciplines (e.g., pulmonologists) was mentioned, as well as with SPC providers and self-help groups. Furthermore, a notable theme emerged concerning the necessity for psychological and spiritual support to address patients’ emotional and existential challenges.

## Standardization of care paths and structures

Statements allocated to this category comprised demands for the standardisation of several aspects in ALS centres themselves. Particularly, staffing with a fix set of professions was named. Consequently, standardised requirements should enable smoother care for both plwALS and providers (e.g. concerning the supply with medical aids or off-label-therapies). The need for care by a team that is both specialised and multiprofessional was emphasised, as well as the need to coordinate all in-house and external team members. Another sub-category comprised statements referring to a double supply gap concerning SPC provision for plwALS: first, health insurances or providers themselves sometimes reject patients as having “not yet a need” for SPC, despite referral by an ALS specialist. This can lead to a serious gap in care, especially when the disease progresses and plwALS are no longer physically capable of attending the ALS outpatient clinic. Secondly, the interviewees stated that the neurological expertise of some SPC providers was limited.

### Overriding healthcare system issues

In addition to issues that directly impact the operations of ALS outpatient clinics, overarching challenges within the healthcare system have been identified to impede the provision of needs-based ALS care. Delays in the provision of medical aids and the inadequate availability of specialized remedies (e.g., physiotherapy, speech therapy) and psychologists for patients with complex neurological disorders were specifically mentioned. The overall inadequate reimbursement of time expenses and of communication-intensive counselling was identified as a leading obstacle in the provision of needs-based care for severely and incurably ill patients.

## Discussion

This interview study aimed to investigate expert neurologists’ perspectives on the structural framework of ALS care in Germany and its clinical implications. Our findings indicate that guideline-based care for plwALS and their families is not guaranteed, even in specialized outpatient centres, because the structural constraints of the healthcare system seem unable to meet the specific needs of these patients—presumably reflecting a limited awareness of these needs. A major barrier are limited financial resources. It became evident, that due to a lack of uniform standards, time resources and staffing vary considerably between centres. This leads to insufficient time resources and inadequately staffed multiprofessional teams in a part of the centres, or recourse on resources originally not intended for ALS care. Further, collaborations with SPC providers are restricted. Psychosocial professionals are typically available only through special, mostly donation-based programs. Consequently, essential aspects of ALS care—such as ACP, psychosocial support, SPC, and socio-medical counselling—are, according to the experts’ perceptions, often inadequately addressed. The discrepancy between high demands on ALS care and the pressure that providers face due to personnel and time constraints risk diminishing provider satisfaction and increasing burnout [32] and, above all, may have far-reaching consequences for patients and their families:

ACP is doubtlessly considered a fundamental component of ALS care and has the potential to strengthen the sense of control of plwALS and alleviate fears of over- and mistreatment. Some patients suffer from existential fatigue or express a desire to hasten death by physician-assisted suicide or euthanasia [33] – sensitive handling of these sentiments is imperative. It is important to note, that discussions on ACP, the terminal phase and dying have to be conducted repeatedly throughout the disease course, and essentially without time pressure. Insufficient resources, as reported by a part of our interviewees, may result in the failure to achieve these requirements and objectives [5].

A multitude of psychosocial and existential stressors that are typical of plwALS, such as fear of loss of control, feeling of being a burden to the family, and experiencing a loss of meaning are, in principle, amenable to psychosocial interventions and spiritual support [34, 35]. Although it is difficult for outsiders to imagine, it is fundamentally possible to adapt to ALS and find meaning and well-being, even with extensive physical limitations and up to a locked-in-state where only eye movements remain [36–38]. Providing psychosocial and spiritual care in order to strengthen patients’ coping abilities has the potential to mitigate a significant component of their suffering. Support through social networks and self-help associations can offer psychological relief by creating a non-judgmental environment and opportunities for sharing experiences [39]. In cases of marked psychological stress or manifest depressive or anxiety disorders, such support alone is insufficient. However, availability of psychologists with expertise in ALS is very limited: none of the interviewed centers included a psychologist as part of the ALS care team. Several interviewees explicitly emphasized the need for psychologists to be readily accessible to patients and their families, either as members of the ALS team or as external consultants. Community-based psychotherapists are difficult for plwALS to access in a timely manner and usually lack expertise with palliative issues. As a result, guideline recommendations to organize professional psychosocial support are challenging to implement in practice. This sharply contrasts with the current state of research and the availability of psychological support for cancer patients in Germany, comprising a wide network of in- and outpatient psycho-oncologists, psychosocial counselling services and self-help-groups [40]. A substantial body of research has demonstrated the efficacy of such support in reducing the psychosocial burden experienced by oncological patients across all stages of the disease [41–43].

Deficiencies in socio-medical counselling, e.g., regarding medical aids, were deemed by 44% of our interviewees. Such insufficient counselling and the resulting gaps in provision may negatively affect patients’ QoL and autonomy, a situation further compounded by delays on the part of payers [9, 44, 45]. Although disease-related and sociodemographic parameters are more frequently analysed [46, 47], studies indicate that insufficient support with socio-medical and organizational tasks further increases caregiver burden [17, 48]. Much has been written about the dual role of caregivers of pwALS, as both affected individuals and primary caregivers, their burden and ethical dilemmas [49], and the urgent and undeniable need to support and involve them closely [4, 17, 47, 50–52]. Unfortunately, our findings indicate that, at least in some of the specialized ALS centers, insufficient capacities are available to conduct family consultations to an appropriate extent.

Awareness of the importance of holistic palliative care for plwALS has increased in the recent years. Many ALS specialists have additional training in palliative care. By definition, they provide primary palliative care from the time of diagnosis, and are highly aware of the evolving needs in terminal disease stages. ALS centres may reach their limits particularly in case of high or complex needs, as reported by our interview partners: a qualification in palliative care is not mandatory and independent from this, the level of expertise varies; time resources are limited, in particular, 24/7 availability and home visits are not feasible; cost-effective compensation for the increased expenditure of time is not given; and psychosocial professionals are not consistently available. Altogether, this hampers an optimal coverage of palliative care needs, which makes the involvement of SPC providers advisable (see also [31]). However, several problems addressed by our interview partners remain to be solved in this field: (1) lack of established predefined criteria for the integration of SPC in ALS care; (2) networking and collaboration with SPC providers are not standardised; (3) resource constraints on the part of SPC providers or a lack of understanding on the part of payers lead to rejections of SPC provision; (4) the legal framework for SPC varies between federal states; and (5) neurological expertise varies widely between the SPC teams, as they still primarily care for oncological patients [53]. Currently, these issues lead to a broad variability of patient journeys and outcomes [25]. It is highlighted in the guidelines, that there is a need for establishing and providing specialized *neuro*palliative care (NPC) in order to meet the specific needs of *neurologically* ill patients [23, 54]. In practice, specialised NPC structures are currently largely not available, care structures in Germany lag significantly behind the guidelines. Pilot projects, namely ALS homecare [55] and TANNE [56], are only temporarily financed.

The aforementioned deficits pertaining to ACP, psychosocial and social medicine can, in principle, be addressed through various approaches. These include the allocation of additional time per patient and visit for neurologists, or the enhanced delegation of these tasks to other professional groups. Both approaches were proposed by half of the interviewed experts. In accordance with the guideline recommendation to establish a multiprofessional team, an *integrative* or a *collaborative approach* are conceivable options, with reference to the integration of psychosocial professions, as well as NPC specialists. The former approach refers to integration into the team, while the latter refers to structured, close collaboration between ALS specialists and external actors from the aforementioned professional groups [8, 57]. Irrespective of the approach chosen, it is imperative to establish explicit standards and guarantee their effective translation into practice through appropriate remuneration. The complementary utilisation of telehealth technologies has the potential to facilitate the implementation of psychosocial care as well as NPC by offering flexibility with regard to location and time, and cost-effectiveness [58, 59]. From the perspective of NPC experts, ensuring equitable access to personalized, needs-based SPC for all patients who require and desire it is essential to guarantee adequate symptom control and to safeguard dignity and autonomy. However, current gaps in care continue to leave many individuals with neurological disorders without appropriate support [60]. Although our project focused exclusively on ALS, these considerations likewise apply to other poor-prognosis neurological disorders, such as Progressive Supranuclear Palsy or Huntington’s disease [61].

The significance of our results is limited by the fact that the interviews were conducted on a convenience sample of experts from specialized ALS centres, which may have introduced selection bias. The interviewees represent a high level of expertise derived from the care of approximately 1,000 plwALS in Germany each year and are thoroughly familiar with both clinical guidelines and patients’ needs. As providers directly involved in the care of plwALS, participants have a professional interest in advocating for the implementation of guideline- and needs-based care and improved structural resources. Further, the findings are based on subjective assessments rather than objective measures, e.g. with regard to time allocation and resource availability, which may limit precision. The participating specialized ALS centres reflect only a segment of the German ALS care landscape. However, it is reasonable to assume that smaller outpatient clinics and practicing neurologists face similar or even greater structural challenges, especially in terms of time and personnel resources. Another limitation is the primary focus on psychosocial and palliative aspects rather than the full spectrum of ALS care. Additional burdens, such as navigating home care, and specific medical diagnostics and treatments, were beyond the study’s scope.

## Conclusions

The present study reveals a marked discrepancy between the requirements for multidimensional, needs-driven and guideline-based care for plwALS and their families as well as for a lack of standards concerning time and personnel resources, team multi-professionalism, and collaboration with other disciplines. While extensive literature underscores the need for multidimensional care and needs-based integration of SPC for plwALS, at least some of the highly specialized ALS centres in Germany are still insufficiently equipped. Although personal commitment and donation-based support can partially offset these shortcomings, there remains an urgent need to improve remuneration and to standardize care structures and networks in ALS care, in order to prevent adverse consequences for those affected. The rising number of ALS diagnoses underscores the necessity for ALS centres to be equipped with the capacity to deliver and coordinate comprehensive, flexible care for plwALS and their families at every stage of the disease [62].

### Contacts

This study was initiated at the University Hospital Carl Gustav Carus at Technische Universität Dresden. PD Dr. med. habil. René Günther and Dr. rer. medic. Katharina Linse are PIs of the study. The project is funded by a seed grant of the ALS association.

## Supplementary Information

Supplementary Material 1

## Data Availability

The data will be deposited on a protected server of the University Hospital Carl Gustav Carus at Technische Universität Dresden, access is strongly regulated. Upon reasonable request including a methodologically sound proposal for the usage of data, data may be shared. Reference lists and individual participant data, as well as the recordings of the interviews will not be shared.

## Abbreviations

ACP: Advance care planning
ALS: Amyotrophic lateral sclerosis
NPC: Neuropalliative care
plwALS: People living with ALS
QoL: Quality of life
SPC: Specialised palliative care
