# Supplementary material for: Between guidelines and reality: expert neurologists’ perspectives on structural resources for ALS care in Germany
Source: Neurol Res Pract. 2026 Apr 8;8(1):22. doi: 10.1186/s42466-026-00481-9 (PMC13063594; doi:10.1186/s42466-026-00481-9)
Supplement: Supplementary file 1 — Supplementary Material 1 [file 42466_2026_481_MOESM1_ESM.docx]

**Supplementary material**

First section of the interview: Questionnaire to descriptively assess quantifiable aspects of local ALS care structures

**SURVEY on care structures in ALS outpatient clinics**

1. Who provides organizational support for the preparation, execution, and follow-up of the ALS consultation (scheduling appointments, requesting documents, assessments, etc. )? (Multiple answers possible)

Ambulance nurse/medical assistant

Case manager

Patient navigator

Social worker at the clinic

Psychologist at the clinic

Chaplain of the clinic

DGM employee

2. How much time do the other parties listed under 1. invest in total per patient and visit for this organizational support (including preparation and follow-up)?

minutes

3. Who regularly provides you with professional support in conducting the ALS consultation and/or in the further care of the patients? (Multiple answers possible)

Ambulance nurse/medical assistant

Case manager

Patient navigator

social worker at the clinic

Psychologist at the clinic

Chaplain of the clinic

DGM employee

Palliative care outpatient clinic/service of our hospital

4. How much time do the other parties listed under 3. invest per patient and visit for this (including preparation and follow-up)?

minutes

5. How much time do YOU invest per patient in consultations (follow-up appointments)?

minutes

6. How much additional time do you estimate to spend on average for preparation and follow-up (prescriptions/orders, reports, telephone contacts, etc.) per patient and visit?

minutes

7. In your view, are the personnel resources you currently have available sufficient (considering the complex, dynamic, multidimensional needs of the patients)?

Yes

no

Yes, but I am already drawing on personnel resources that are not strictly intended for the ALS consultation.

No, and I am already drawing on personnel resources that are not strictly intended for the ALS consultation.

8. In your view, are the time resources available to you and, if applicable, your team sufficient (considering the complex, dynamic, multidimensional needs of the patients)?

Yes

no

Yes, but it actually exceeds my/our available time.

No, and it already exceeds my/our available time.

9. Is the remuneration sufficient to cover costs in view of the services rendered?

Yes

no

10. If you consider the available personnel resources and/or time to be insufficient: which areas of responsibility do you sometimes neglect due to time constraints (measured against the needs of the patients)? (Multiple answers possible)

I feel the available personnel resources and time are sufficient

to update the medical history.

update neurological status

medical treatment of symptoms (e.g., increased salivation, sleep disturbances,... )

give more information about ALS

advice and information on medical aids

advice and information on social law matters (e.g. degree of disability, care level, pension, care and support services,,... )

Discussions about treatment/advace care planning (what options are available, inquiring about the patient's wishes and values, including advice on advance directives and powers of attorney)

discussions on psychological strain (anxiety, feelings of helplessness and hopelessness)discussions about issues concerning death/dying (weariness of life or fear of dying; passive or indirect euthanasia, physician-assisted suicide, palliative care options at the end of life)

discussions on social strain (e.g. conflicts regarding care situations, medical measures, burden on relatives)

discussions on existential / spiritual strain (meaning of life and dignity, reflections on death)

conversations with next of kin

other, namely...

11. Does your outpatient clinic have any collaborations with specialized palliative care services (SAPV teams, palliative care units, hospices)?

no, not at all

No, only very loose contacts; I merely recommend that patients contact these services.

Yes, there is a loose cooperation; I refer my patients to these services and we exchange reports.

Yes, there is close cooperation with regular case conferences/phone calls, if necessary.

12. Do you usually care for "your" patients until their death?

Yes, usually contact is maintained at least by telephone / video visit / via SAPV services until death.

No, usually patients no longer come to the consultation once their illness reaches a certain severity and contact is lost.

13. What suggestions do you have for how patient care in ALS clinics could be improved? (Multiple answers possible)

I see no need for improvement.

more time available for me (and my team) per patient

better reimbursement of the actual expenditure of time

further professions in team, specifically...

better networking with other medical professionals/services (pulmonologists/clinics, nutritional counseling/medicine, outpatient palliative care services, palliative care units and hospices,... )

home visits are possible (legal issues, time + compensation)

conversations with next of kin are possible (time + compensation)

facilitated access to relevant training courses ((Neuro-)Palliative Care / Palliative Medicine, communication skills, respiration/ventilation, assistive devices,... ) for me and my team

other, namely...

End of form
